# A functional trait perspective on restored temperate grassland responses to changing winter insulation and managed disturbance by fire

**DOI:** 10.1002/ajb2.70109

**Published:** 2025-10-09

**Authors:** Katherine T. Charton, Jonathan J. Henn, Michelle A. Homann, Christopher R. Warneke, Ellen I. Damschen

**Affiliations:** ^1^ Department of Integrative Biology University of Wisconsin‐Madison Madison WI USA; ^2^ Institute on the Environment University of Minnesota‐Twin Cities St. Paul MN USA; ^3^ Institute of Arctic and Alpine Research University of Colorado‐Boulder Boulder CO USA

**Keywords:** adaptive management, biodiversity, climate change, cold tolerance, plant community dynamics, prescribed fire, resist‐accept‐direct, restoration outcomes, snow, tallgrass prairie

## Abstract

**Premise:**

Understanding how disturbance regimes influence temperate grasslands is crucial for adapting management strategies to climate change, particularly in response to the loss of winters. The interaction between disturbance and climate can alter winter soil insulation and potentially the plant community. Examining the role of functional traits in determining community outcomes can help inform whether grasslands will remain resilient to climate change or whether management will need to be adapted proactively.

**Methods:**

We analyzed 7 years of data from a restored temperate grassland experiment to assess how the interaction between management type and timing (i.e., unmanaged control, spring burn, fall burn, and fall mow) and winter snow manipulations (i.e., snow reduction, snow control, and snow addition) affects plant community composition and whether functional traits are related to community turnover.

**Results:**

Changes in the plant community were driven mainly by management type and timing, with minimal influence from winter snow manipulations. While greater stress tolerance was associated with colonization when winter soil insulation was low, overall functional traits had a relatively minor relationship with community turnover.

**Conclusions:**

The minimal effects of winter snow manipulations, combined with the community's shift toward stress‐tolerant strategies when winter soil insulation was low, suggest that grasslands may be resilient to winter snow loss in the short term. However, limited colonization by species that are not stress tolerant could drive local extinctions over time. Management strategies that support colonization and retain soil insulation, such as spring burns that maintain disturbance while preserving insulating litter, may help prevent longer‐term impacts.

Climate change is altering ecosystems worldwide, but some of its most profound and underappreciated effects occur in winter. In temperate regions, climate is changing more rapidly in winter than in other seasons (IPCC, 2013). Warming winter temperatures are leading to more precipitation falling as rain rather than snow, while increased temperature variability allows any accumulated snow to melt intermittently, resulting in overall reductions in snow accumulation (Notaro et al., 2014, 2015). Snow acts as an insulator, providing thermal protection to the soil, so despite winters having on average higher air temperatures, soil temperatures can actually be lower in winters with reduced snow accumulation (Brown and DeGaetano, 2011; Pauli et al., 2013). These shifts are particularly concerning for perennial‐dominated ecosystems like temperate grasslands, where belowground plant organs such as buds and roots are critical for overwintering and regeneration (Lubbe et al., 2021). Loss of winter snow cover may increase exposure to freezing events and cold stress (Lubbe et al., 2021), with potential ramifications for temperate grassland plant communities and their stability (Kreyling et al., 2019).

To determine how best to conserve and restore temperate grasslands facing the loss of winters, it is necessary to assess the effects of different management actions on community outcomes and their alignment with conservation goals (Brudvig and Catano, 2021; Lynch et al., 2022; Magness et al., 2022). As snow cover diminishes, grassland ecosystems may become increasingly shaped by the type and timing of disturbance regimes, which modify another important component of overwintering insulation: the litter layer. Low‐intensity, frequent fire has historically been a key disturbance in temperate grasslands (Bond and Keeley, 2005; Bond et al., 2005) and serves as an important management tool to maintain plant community quality and diversity (Bowles and Jones, 2013; Larson et al., 2020; Henn and Damschen, 2022). The timing of fire, however, also influences the presence of dead plant litter, which affects winter soil temperatures (Lubbe and Henry, 2019; Henn and Damschen, 2022). For instance, removing litter before winter through fall burns exposes plants to damaging cold when snow accumulation is low, whereas spring burns allow litter to overwinter, thereby increasing winter soil temperatures (Lubbe and Henry, 2020; Henn and Damschen, 2022). Mowing is sometimes employed as an alternative to fire (Davison and Kindscher, 1999) but differs in its ecological effects due to the absence of smoke, heat, and litter consumption (Randa and Yunger, 2001; Kitchen et al., 2009).

Temperate grassland plant communities are likely influenced by the unique insulative conditions resulting from the interaction between loss of winter snow and management type and timing (Figure 1), which must be considered in decision making. The resist–accept–direct (RAD) framework (Lynch et al., 2021; Schuurman et al., 2022) provides a structured approach to guiding ecosystems through varying degrees of transformation, especially under uncertain and context‐dependent outcomes (Heller and Zavaleta, 2009; Crausbay et al., 2022). In this context, the resist pathway involves selecting the appropriate management type and timing to preserve the existing plant community and its associated functions as winter conditions shift. In contrast, the accept pathway entails continuing current management practices without modification, resulting in either the maintenance of the community or its divergence, depending on the resilience of grassland species in response to altered winter conditions. Finally, the direct pathway focuses on intentionally modifying management type and timing to promote community divergence under novel winter conditions, facilitating a shift toward different plant communities that may be better suited to the changing climate. If the goal is to maintain grasslands as grasslands, considering their current quality and diversity, a key question is whether management actions need to be adapted to the new climate to preserve existing plant communicates (i.e., resist), or whether the community is inherently resilient, making such adaptations unnecessary (i.e., accept).

**Figure 1:**
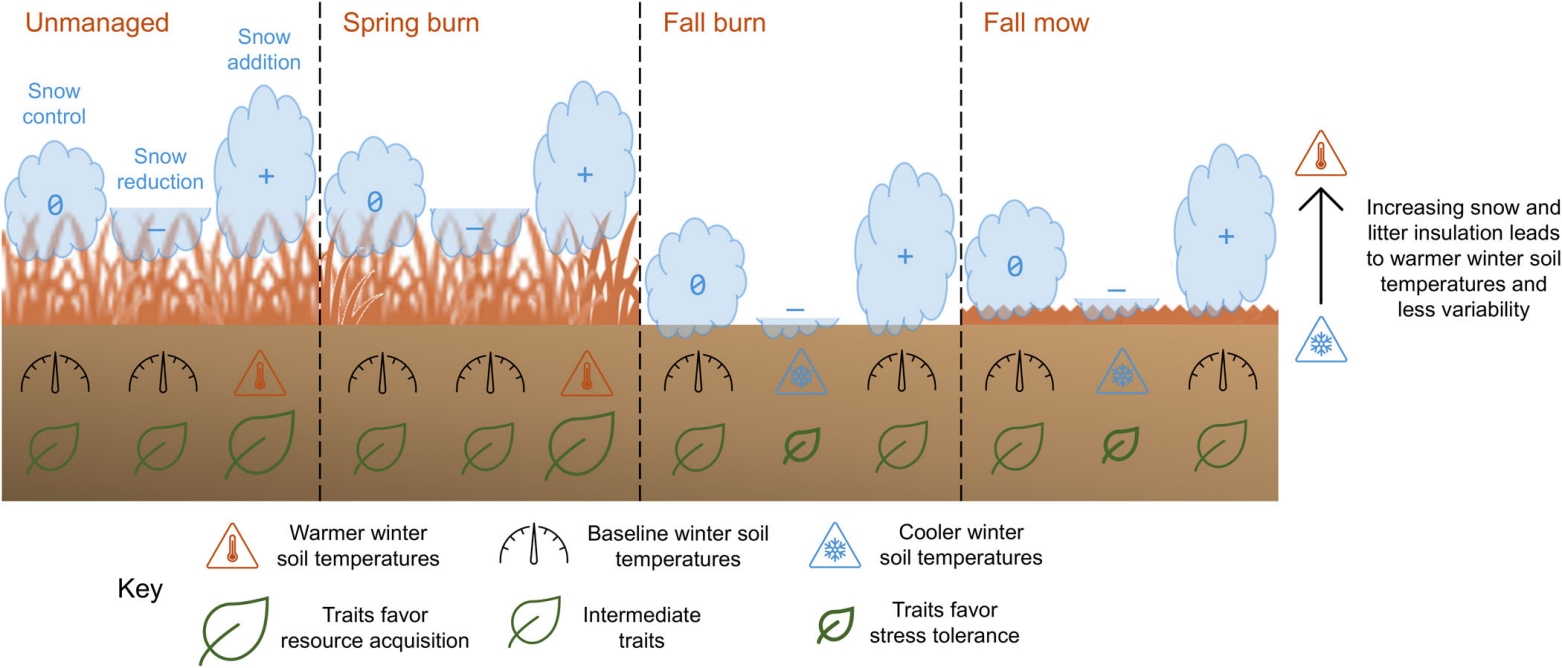
Effects of management type and timing and winter snow manipulations on snow and litter insulation during winter and predicted associations with plant functional trait syndromes.

Understanding the effects of management outcomes on grassland communities under climate change is complicated by species‐specific responses and the longevity and persistence of species within perennial‐dominated ecosystems, making it difficult to detect changes over short time scales (Symstad et al., 2003; Hughes et al., 2017). Functional traits, however, offer a valuable lens independent of individual species identities for assessing community turnover and make it possible to detect change on shorter timescales than might otherwise be apparent in whole‐community dynamics (Lavorel and Garnier, 2002; Webb et al., 2010). Functional traits are measurable characteristics of plants that can serve as a proxy for fitness and influence how species interact with their environment and respond to stressors (McGill et al., 2006; Violle et al., 2007; Shipley et al., 2016). For example, fire, a stressor in its own right, has driven the evolution of fire‐adapted plant communities with traits such as specialized belowground organs and the ability to resprout after disturbance (Lavorel and Garnier, 2002; Archibald et al., 2019). In the absence of fire, grassland plant communities can undergo significant shifts, favoring species that are less fire‐adapted because fire‐related traits are no longer selected for in the absence of this disturbance (Alstad and Damschen, 2015; Alstad et al., 2016). This trait‐based approach provides insight into which collections of traits are likely to persist or decline and how communities might reassemble under new climatic conditions and differing disturbance regimes (Gondard et al., 2003; Mouillot et al., 2013). Functional traits can therefore offer predictive power for operationalizing decision‐making frameworks for management and restoration.

Trait syndromes, that is, sets of co‐occurring traits, offer deeper insights into how plant communities assemble than single traits alone (e.g., Reich et al., 2003; Kraft et al., 2015; Raffard et al., 2017). In temperate grasslands, many species exhibit co‐tolerance, possessing traits that confer resilience to both fire and cold (Ladwig et al., 2018). For instance, species capable of resprouting after fire often have adaptations that also protect against cold stress, such as deeper bud structures (Lavorel and Garnier, 2002; Archibald et al., 2019). Co‐tolerance may enable grasslands to remain resilient to the effects of climate change and loss of winter snow because many grassland species that are already tolerant to fire may also exhibit co‐tolerance to increasingly cold winter soil temperatures. In general, however, stress tolerance represents a trade‐off with resource acquisition (Wright et al., 2004; Díaz et al., 2016). Stress‐tolerant species, characterized by slower growth and greater investment in tissue durability, are better suited to survival in environments with harsh or stressful conditions, while resource‐acquisitive species tend to have more vulnerable, shorter‐lived tissues but faster growth and reproduction, making them more likely to establish and persist when conditions are mild (Westoby, 1998; Adler et al., 2014). Whether selection for stress tolerance over resource acquisition is sufficient for the community to remain resilient to loss of winters across various disturbance regimes remains to be tested.

To inform how grassland management can be adapted to climate change, we leveraged 7 years of data from a field experiment to assess (1) how the interaction between management type and timing (i.e., unmanaged control, spring burn, fall burn, and fall mow) and winter snow manipulations (i.e., snow reduction, snow control, and snow addition) influences the plant community in a restored temperate grassland and (2) whether functional traits, particularly those related to stress tolerance versus resource acquisition, are associated with community turnover. We hypothesized that (1) any management action (i.e., spring burn, fall burn, or fall mow) would increase biomass, richness, and diversity, but that low insulation (i.e., fall burn or mow and/or snow reduction; Figure 1) would decrease these metrics, potentially offsetting the benefits of management. Additionally, we hypothesized that (2) stress‐tolerant species would increase in abundance and colonization and decrease in extinction in response to any management action (i.e., spring burn, fall burn, or fall mow) and/or low insulation (i.e., fall burn or mow, and/or snow reduction; Figure 1), while resource‐acquisitive species would increase in abundance and colonization and decrease in extinction in response to lack of management (i.e., unmanaged control) and/or relatively high insulation (i.e., spring burns and/or snow control or addition; Figure 1).

## MATERIALS AND METHODS

### Experimental design

**Figure 2:**
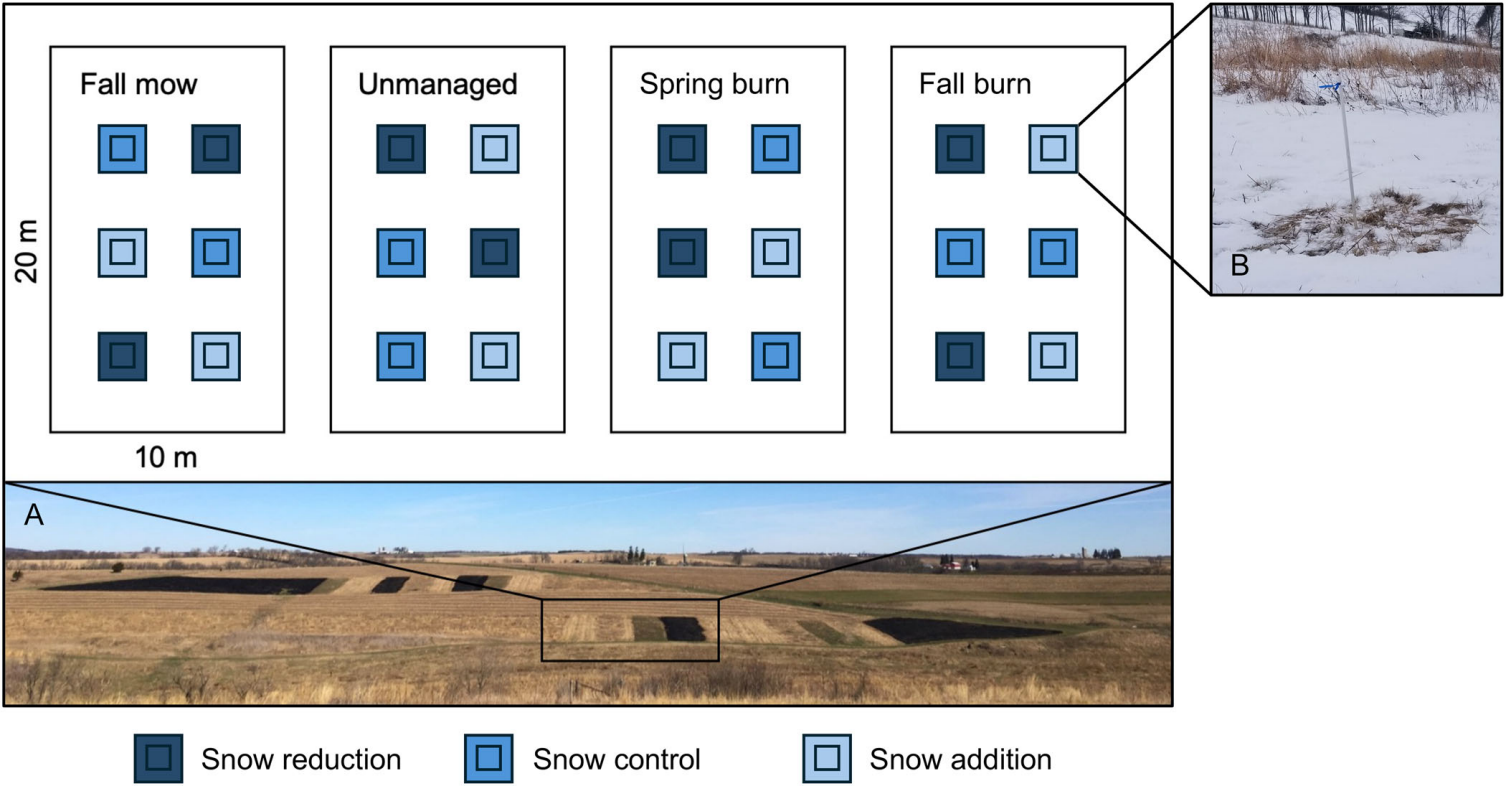
Experimental design at Mounds View Grassland. (A) Within each experimental block, there are four nested management type and timing treatment plots (i.e., unmanaged control, spring burn, fall burn, fall mow). Within each plot, there are six winter snow manipulation subplots (B shows one subplot). Each treatment level (i.e., snow reduction, snow control, and snow addition) has two replicates. Within each subplot is a further nested subplot in which vegetation is sampled.

We conducted a long‐term, fully factorial field experiment to investigate the interaction between management type and timing and winter snow manipulations on restored temperate grassland plant communities. The experiment was set up in 2016 at Mounds View Grassland in Iowa County, WI, USA (42.95807 N, 89.86454 W) in areas restored to grassland in 2011 (Henn and Damschen, 2022). Within this restoration, we created eight experimental blocks, each containing four 10 × 20 m plots separated by 3‐m mowed buffers that also served as burn breaks. These plots were randomly assigned to one of four management type and timing treatments (i.e., unmanaged control, spring burn, fall burn, and fall mow; Figure 2). Nested within each management plot, we established six 2 × 2 m snow manipulation subplots arranged in a grid with 2‐m spacing between them. Subplots were randomly assigned to one of three winter snow manipulation treatment levels (i.e., snow reduction, snow control, and snow addition; Figure 2), with two replicates per management treatment plot, totaling 192 subplots across the experiment. We measured plant community responses in a central 1 × 1 m area of each subplot to minimize edge effects at the 2 × 2 m subplot level (Figure 2).

### Treatment applications

After the growing season in which the experiment was established, fall mow treatments were applied annually in mid‐fall (i.e., October–November), fall burn treatments occurred annually in late fall (i.e., November–December) approximately 1 month after the fall mow, and spring burn treatments were applied annually in mid‐spring (i.e., April; Appendix S1: Table S1). Unmanaged control treatments remained undisturbed for the duration of the study. Spring and fall burn treatments reduced litter depths (8.9 ± 0.5 cm and 7.7 ± 0.6 cm, respectively), but only the effects of the fall burn treatments were expected to persist into the winter because litter likely reaccumulates after spring burns, while fall mow treatments lead to intermediate litter depths relative to unmanaged treatments (1.9 ± 0.6 cm; Figure 1; Appendix S1: Table S2). Winter snow manipulations were implemented each time more than 10 cm of snow accumulated at the site during winter (i.e., December–February; except for the 2019–2020 winter when limited staffing prevented snow manipulation). Consequently, the total snowfall and the number of snow manipulations varied by year (Appendix S1: Table S1). In the snow reduction treatments, snow depth was reduced to approximately 2 cm above the soil by shoveling, and this snow was added to the snow addition treatments, while snow control treatments were left unmanipulated. Snow reduction treatments reduced snow depths over the winter (15.5 ± 0.4 cm), while snow addition treatments increased snow depths relative to snow control treatments (24.6 ± 0.5 cm; Figure 1; Appendix S1: Table S2).

### Soil temperature data collection

To monitor treatment effects on soil temperature, Thermochron iButton data loggers (iButtonLink Technology, Whitewater, WI, USA) were placed in the center of a subset of the subplots stratified by treatment combination each winter. The iButtons were waterproofed in small zip‐lock bags and buried 2 cm below the soil surface after completing fall burn and mow treatments and remained in place until planning for the spring burn treatment commenced (i.e., December to February each year). Temperature readings were recorded every 2 h. From these data, we calculated the average minimum and maximum temperatures for December, January, and February (DJF) for each subplot.

### Plant community data collection

We assessed the plant community annually at the end of the growing season for 7 years (i.e., August–September 2017–2023; except for the 2020 growing season when limited staffing resulted in subsampling half the plots). Percentage cover and maximum height of all plant species within each inner 1 × 1 m subplot were visually estimated, and species overlap was allowed, so total cover could exceed 100%. In the first two growing seasons, we used Daubenmire cover classes (Daubenmire, 1959) and assigned observations to the midpoints of each class. From the third season onward, we estimated cover to the nearest percentage for values > 1% and to the nearest tenth for values < 1%. Sampling aligned with peak flowering period, coinciding with peak productivity and facilitating accurate species identification. We included all taxa with more than one observation across the 7 years of data in the analysis, for a total of 65 observed taxa, 59 of which were identified to species and five to genus (Chadde, 2013; Appendix S1: Figure S1). We use “species richness” hereafter to refer to the number of observed taxa, regardless of the level of taxonomic resolution.

### Functional trait data collection

We assessed a suite of functional traits known to reflect stress tolerance and resource acquisition trade‐offs for each species identified in the community data, including plant height, leaf area, specific leaf area (SLA), leaf dry matter content (LDMC), foliar nitrogen content (N), leaf tissue cold tolerance (LT_50_), and dispersule mass (Appendix S1: Table S3). Plant height and leaf area are commonly associated with competitive ability for light and photosynthetic capacity, but taller plants and larger leaves require greater resource investment and may be more vulnerable to environmental stress (Westoby, 1998; Adler et al., 2014; Díaz et al., 2016). Specific leaf area and LDMC capture trade‐offs between tissue durability and growth rate, with high SLA and low LDMC associated with fast growth and less robust tissues, and low SLA and high LDMC associated with more durable leaves and enhanced stress tolerance (Wright et al., 2004; Díaz et al., 2016). Nitrogen reflects metabolic activity and photosynthetic capacity, with high N linked to acquisitive strategies and low N associated with conservative, stress‐tolerant resource use (Wright et al., 2004; Díaz et al., 2016). Leaf tissue cold tolerance provides a direct measure of tissue sensitivity to freezing and represents an axis of environmental filtering particularly relevant in climates with variable winter conditions (Raunkiær, 1934). Finally, dispersule mass reflects a trade‐off between dispersal potential and establishment success, where smaller propagules tend to disperse more widely but establish less reliably, and larger propagules often enhance establishment and support more stress‐resilient seedlings (Westoby, 1998; Adler et al., 2014; Díaz et al., 2016).

All measurements were taken from visually healthy, mature plants during the growing season across grasslands in southern Wisconsin over the past decade (i.e., 2013–2023) and averaged from at least five individuals per species. We measured plant height in the field as the shortest distance from the highest photosynthetic tissue to the ground. In the lab, fresh leaves collected in the field were weighed, scanned using a flatbed scanner, and analyzed for projected leaf area using ImageJ (Schneider et al., 2012) with the LeafArea package 0.1.8 (Katabuchi, 2015) in R 4.3.1 (R Core Team, 2023). After drying for a minimum of 2 weeks at 40°C, leaves were reweighed to determine dry mass, allowing us to calculate SLA (i.e., one‐sided leaf area/oven‐dry mass) and LDMC (oven‐dry mass/fresh mass; Pérez‐Harguindeguy et al., 2016). Dry leaf tissues were analyzed for N at the Stable Isotope Ecology Laboratory at the University of Georgia, Athens, GA, USA. Leaf tissue cold tolerance, defined as the temperature at which 50% of the leaf tissue was damaged (Zhang and Willison, 1987), was measured by assessing electrolyte leakage from the tissue after exposure to cold treatments in an incubator (VWR, Radnor, PA, USA), using established protocols (Murray et al., 1989; Ehlert and Hincha, 2008; Ladwig et al., 2018). Leaves for LT_50_ were collected early in the growing season (i.e., May–July), when they were young and fully expanded. Although LT_50_ values measured early in the growing season may not capture the full extent of cold acclimation, they offer a standardized and ecologically relevant comparison of interspecific cold tolerance under consistent physiological conditions, analogous to other standardized collection strategies that enable comparisons across time and space despite known, unmeasured sources of variation (Pérez‐Harguindeguy et al., 2016). “Dispersule” is defined as the plant structure responsible for dispersal, which may contain one or more seeds, depending on the species (Hintze et al., 2013). We measured dispersule mass immediately after collection for fleshy‐fruited dispersules and after air drying for all other types. Trait data were available for 89% to 98% of taxa and represented 86% to >99% of total community abundance, except for LT_50_, which was only available for 50% of taxa but still covered 83% of community abundance (Appendix S1: Table S3). We did not supplement missing data with external sources to preserve the representation of local adaptation. While incomplete trait data may affect trait‐specific interpretations, it is unlikely to bias overall community‐weighted patterns given the high coverage for community abundance.

### Community change analyses

To examine how management type and timing interact with winter snow manipulations to affect the plant community, we calculated the change (i.e., response ratio; Hedges et al., 1999) in several metrics averaged over the first 3 years of data (i.e., 2017–2019) and the most recent 3 years (i.e., 2021–2023). Metrics included estimated biomass (i.e., total plant volume or the summation of percentage cover × maximum height; Catchpole and Wheeler, 1992), species richness, and Shannon's diversity (Shannon, 1948; Good, 1953), the latter two calculated using the R package vegan 2.6‐4 (Oksanen et al., 2022). We fitted linear mixed effects models for each response using the R package lme4 1.1.35.1 (Bates et al., 2015), including management type and timing (i.e., unmanaged control, spring burn, fall burn, and fall mow), winter snow manipulations (i.e., snow reduction, snow control, and snow addition), and their interaction as fixed effects, along with a random intercept for nested subplots within plots within blocks. We also considered minimum and maximum DJF soil temperatures as predictors in place of the treatment categories using the same model formulation to capture the direct environmental conditions that result from the treatments. To visualize the influence of the treatments on changes in relative abundances, we used nonmetric multidimensional scaling (NMDS) in the vegan package. The increase or decrease in relative abundance of each species was quantified by comparing community composition between the two time periods (i.e., 2017–2019 and 2021–2023). To assess differences among treatments on changing abundance, we performed a permutational multivariate analysis of variance (PERMANOVA), accounting for the blocked structure of the data using the R package survival 5.5‐8 (Therneau, 2024). The first axis primarily reflected differences between experimental blocks (Appendix S1: Figure S2), therefore, the analysis focuses on the second and third axes, which reflected treatment‐driven changes in abundance.

### Functional trait analyses

**Figure 3:**
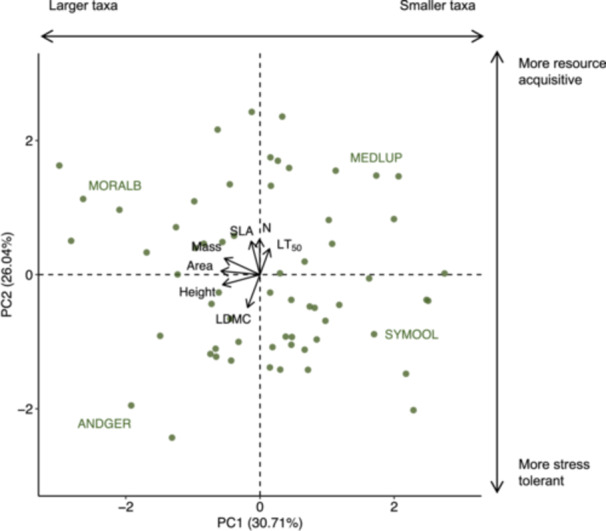
Principal component analysis of seven functional traits of the observed taxa in the community where the second axis (i.e., PC2) reflects trade‐offs between stress tolerance and resource acquisition. Points represent individual taxa, with an example taxon labeled in each quadrant, and vectors represent the loading of each trait. The amount of variance explained by each axis is listed alongside the axis title. Height = plant height, SLA = specific leaf area, Area = leaf area, LDMC = leaf dry matter content, N = foliar nitrogen content, LT_50_ = leaf tissue cold tolerance, Mass = dispersule mass, ANDGER = *Andropogon gerardii* Vitman, MEDLUP = *Medicago lupulina* L., MORALB = *Morus alba* L., SYMOOL = *Symphyotrichum oolentangiense* (Riddell) G.L. Nesom.

To test whether functional traits interact with the treatments to influence changes in the plant community, we again used the change in relative abundance and calculated the number of colonization and extinction events for each species in the community. Colonization was defined as the first appearance of a species in a plot where it had not been previously recorded, while extinction was defined as the absence of a species that had been previously present across the two time periods (i.e., 2017–2019 and 2021–2023). We used the second axis (i.e., PC2) from a principal component analysis (PCA) of the seven measured functional traits (i.e., plant height, leaf area, SLA, LDMC, N, LT_50_, and dispersule mass; Figure 3) as a fixed effect in the models, because PC2 captured the variation in all traits along a singular axis that best reflected the trade‐offs between stress tolerance (i.e., low values of PC2) and resource acquisition (i.e., high values of PC2; Ladwig et al., 2018) rather than taxa size. For the PCA, we log‐transformed leaf area, LMA, LDMC, and dispersule mass to meet assumptions of normality and gap‐filled missing trait data using multiple imputation by chained equations using the R package mice 3.16.0 (van Buuren and Groothuis‐Oudshoorn, 2011). We fitted linear mixed effects models for each response, including the fixed effects of management type and timing, winter snow manipulations, PC2, and their three‐way interaction, along with a random intercept for nested subplots within plots within blocks. Additionally, we ran separate models for each individual trait as predictors. We again also considered minimum and maximum DJF soil temperatures in place of the treatment categories. We ran linear mixed effects models for changes in abundance and generalized linear mixed effects models with a binary distribution for colonization and extinction events using the lme4 package. From these analyses, we calculated standardized effect sizes analogous to Cohen's *d* for each predictor (Hedges, 2007; Westfall et al., 2014).

For all models, categorical predictors were compared to control conditions (i.e., unmanaged and snow controls). Test statistics and *P*‐values were calculated using Laplace approximations for linear mixed effects models and Satterthwaite approximations for generalized linear mixed effects models with the R package lmerTest 3.1.3 (Kuznetsova et al., 2017). We used the R package emmeans 1.8.9 (Lenth, 2022) to test pairwise differences in the estimated marginal means for each treatment combination and the R package rsq 2.6 (Zhang, 2022) to calculate the proportion of variation explained by the fixed effects in each model. Significance was set at *α* = 0.05.

## RESULTS

**Figure 4:**
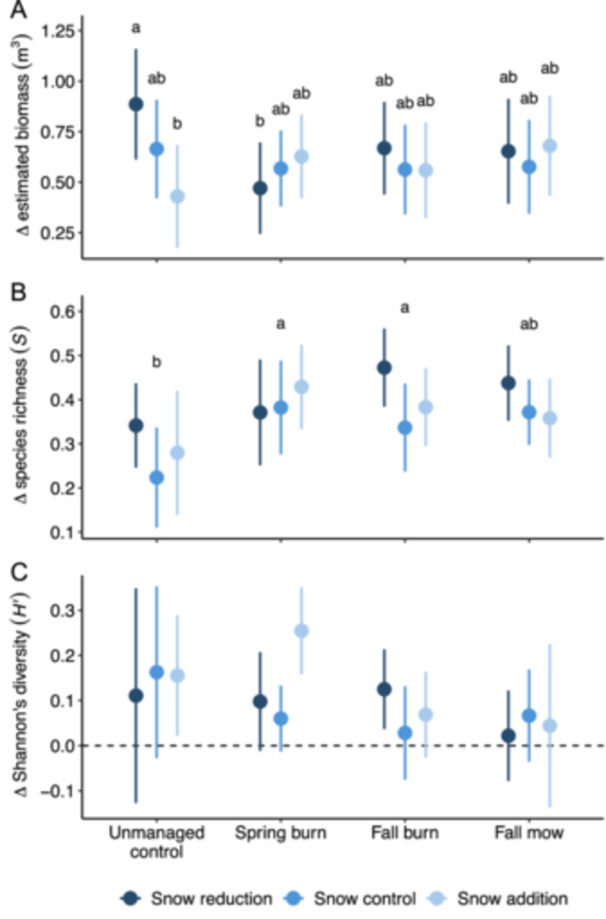
Effects of management type and timing, winter snow manipulations, and their interaction on changes in the plant community, including (A) estimated biomass, (B) species richness, and (C) Shannon's diversity. Points represent group means, and error bars represent 95% confidence intervals. Letters indicate significant pairwise differences in the estimated marginal means of each treatment combination. Note vertical axes are on different scales.

Change in the plant community was primarily influenced by management type and timing rather than winter snow manipulations. Overall, biomass, richness, and diversity increased over the 7‐year study (Figure 4; Appendix S1: Table S4). Species richness increased more with spring and fall burns compared to unmanaged controls (Figure 4; Appendix S1: Table S4). Only estimated biomass was influenced by an interaction between management type and timing and winter snow manipulations, increasing more in unmanaged controls with snow reduction compared to unmanaged controls with snow addition and spring burns with snow reduction (Figure 4; Appendix S1: Table S4). Using winter soil temperature as a predictor additionally revealed that diversity decreased with increasing maximum winter temperature (Appendix S1: Figure S3; Appendix S1: Table S5). Increases in relative abundance varied by management type and timing, while decreases were influenced by both management type and timing and winter snow manipulations (Appendix S1: Figure S4, Table S6).

**Figure 5:**
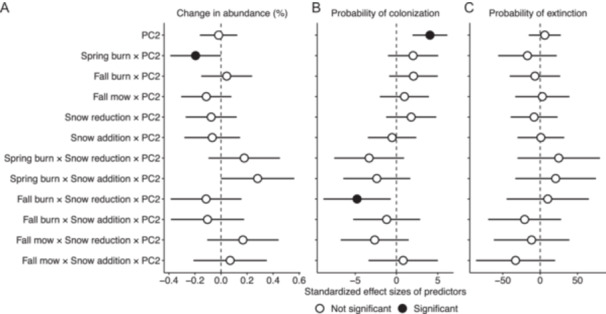
Effects of management type and timing, winter snow manipulations, functional trait syndrome (i.e., PC2; Figure 3), and their interaction on changes in the community, including (A) change in abundance, (B) probability of colonization, and (C) probability of extinction. Low values of PC2 reflect species with greater stress tolerance and high values of PC2 reflect species with greater resource acquisitiveness. Points represent average standardized effect sizes; filled points mean *P* < 0.05; error bars represent 95% confidence intervals. Note horizontal axes are on different scales.

In certain cases, functional traits were associated with community turnover under different management type and timing and winter snow manipulations. Overall, colonization (996 instances of species gained) occurred more frequently than extinction (342 instances of species lost) over the study period. Across the entire experiment, regardless of management type and timing or winter snow manipulations, resource acquisitiveness (i.e., higher values of PC2; Figure 3) was associated with more colonization events (Figure 5; Appendix S1: Table S7). On the other hand, stress tolerance (i.e., lower values of PC2; Figure 3) was associated with increased abundance in spring burns and more colonization events in fall burns with snow reduction (Figure 5; Appendix S1: Table S7). The magnitudes of these effects, however, were relatively small, and community turnover in all other management and climate scenarios appeared unrelated to functional traits, including when considering winter soil temperatures rather than treatment categories (Appendix S1: Figure S5, Table S8).

## DISCUSSION

Our study demonstrated that management type and timing were the primary drivers shaping the plant community in this restored temperate grassland more than winter snow manipulations. Over the 7‐year study, we consistently observed increases in biomass, richness, and diversity, with the greatest increase in richness occurring in response to spring and fall burns compared to unmanaged controls. Although winter snow manipulations had minimal direct influence on these metrics, they interacted with management in specific cases, such as when reduced snow cover led to decreased biomass in spring burns compared to reduced snow cover in unmanaged controls. This finding contradicted the initial hypothesis that reduced insulation from snow would consistently diminish biomass, richness, and diversity thereby negating the positive responses to management. While functional traits were relatively weakly associated with community turnover in only a few cases, those associations aligned with hypothesized trade‐offs between stress tolerance and resource acquisition in relation to management type and timing and winter snow manipulations. Stress‐tolerant species were more likely to increase in abundance in spring burns and colonize in the stressful, low‐insulative conditions of fall burns combined with snow reduction, creating opportunities for otherwise smaller, slower‐growing stress‐tolerant species to establish and persist in these harsh conditions. Meanwhile, resource‐acquisitive species were more likely to colonize across the entire experiment, suggesting that the variability introduced by the experimental treatments, along with the relatively young age of this restoration, may have created opportunities for the establishment and persistence of faster‐growing species in conditions that were less stressful than initially hypothesized.

The positive response of species richness to both spring and fall burns highlights the importance of prescribed fire, regardless of season, in maintaining biodiversity and ecosystem function. These findings align with research indicating that fire clears litter and creates open spaces for new species establishment (Knapp and Seastedt, 1986; Hulbert, 1988). Interestingly, this increase in richness does not extend to overall diversity, suggesting that non‐dominant, disturbance‐dependent species [e.g., *Panicum virgatum* L., *Schizachyrium scoparium* (Nash) E. P. Bicknell] establish more frequently after fire, while a few species maintain higher relative abundance and disproportionately influence community structure (e.g., *Andropogon gerardii* Vitman; Appendix S1: Figure S1). This observation is further supported by the higher frequency of colonization compared to extinction, suggesting a potential extinction debt, that is, a delayed loss of non‐dominant species that may temporarily persist under altered management regimes but ultimately go locally extinct over time (Tilman et al., 1994; Kuussaari et al., 2009). Although the 7‐year duration of this study is longer than many ecological experiments, it may still be insufficient to capture longer‐term (i.e., decadal) turnover in the community.

The minimal effect of winter snow manipulations on the community suggests that, at least within recently restored temperate grasslands, resilience to winter climate changes may be present, independent of management actions. While disturbances like fire have pronounced immediate and direct effects on the conditions plants experience (Zedler, 2007), the consequences of reduced winter snow may unfold over longer durations, exerting subtler influence on community dynamics (Wu et al., 2015). The lack of observed climate effects could be due to these small or delayed impacts but could also be caused by the inadequacy of the snow manipulations to accurately reflect real‐world processes in a rapidly changing climate (Langley et al., 2018). Variability in ambient conditions, such as the recent winters with below‐average snowfall (e.g., 2021–2022; Appendix S1: Table S1), complicates the establishment of accurate controls that reflect long‐term reference conditions. This variability may diminish the informative value of winter snow manipulations compared to those based on average ambient climatic conditions over extended periods (Jentsch et al., 2007).

While the role of functional traits in influencing community dynamics under various disturbance and climate scenarios was evident, the magnitude of their effects on community turnover was relatively minor. This weak influence of traits on community turnover may be due to the adaptations plants have developed over evolutionary timescales to cope with a wide range of climate conditions (e.g., Craine et al., 2012), including variations in winter soil temperature driven by changing insulation. The relatively small fluctuations in winter soil temperature associated with the treatments are unlikely to significantly impact many species. For instance, leaf tissue cold tolerances (LT_50_) in this community ranged from –10.0°C to –6.5°C (Appendix S1: Table S3), but notably, only 52% of the observed minimum winter temperatures fell below the LT_50_ threshold for the least cold‐adapted species, and just 20% fell below that for the most cold‐adapted species. Consequently, the soil temperature fluctuations resulting from winter snow manipulations may not affect species persistence. Plants may also possess the ability to adjust to such environmental variability plastically (Matesanz et al., 2010). For example, the functional traits of young spring leaves have previously been shown to vary with management type and timing such that fall burns led to more stress tolerance within a species and spring burns more resource acquisitiveness, likely reflecting responses to different winter conditions (Henn et al., 2022).

Though beyond the scope of this study, one possible explanation for the minimal effect of winter snow manipulations on the plant community is that cumulative sublethal effects on vulnerable plant life stages, such as seeds or seedlings (Williams et al., 2015; Ósvaldsson et al., 2022), may contribute to extinction debt in the context of altered insulative conditions. Additionally, the relationship of functional traits with community turnover may have been relatively weak due to challenges in selecting traits that accurately reflect the mechanisms driving community responses (Funk et al., 2017; Hagan et al., 2023). Commonly measured traits, while useful for ease of measurement, may not fully capture how plants cope with stress under changing winter conditions. Traits more closely tied to disturbance and cold stress tolerance, such as bud and root tissue cold tolerance, bud bank size, rooting depth, and carbohydrate storage, may provide a better understanding of plant resilience (Klimešová et al., 2008; Ott et al., 2019; Freschet et al., 2021; Nettesheim et al., 2021; Weigelt et al., 2021).

Where functional traits did play a role, the community appeared to possess a sufficient range of stress‐tolerant trait syndromes to maintain resilience under reduced winter insulation. Prior research suggests that species adapted to fire may also exhibit greater tolerance to cold stress (Ladwig et al., 2018), indicating that ongoing management may foster co‐tolerance within the community. This resilience is particularly notable given the relatively young age of the restoration, when early successional stages typically favor resource acquisition as communities develop (Shipley et al., 2006). Our finding that resource acquisitiveness led to more colonization events across the entire experiment supports the idea that resource acquisitiveness is favored in young restorations. Over longer periods, however, stress‐tolerant species may become even more dominant, potentially further enhancing resilience and increasing functional redundancy. Additional research is needed to understand how these dynamics play out over a broader range of temperate grassland environments, considering region, disturbance and land‐use history, and restoration age and quality, among other factors.

### Management implications

The complexity and unpredictability of plant community responses to disturbances and climate variability challenge the restoration and conservation of grasslands worldwide. In the context of the resist‐accept‐direct (RAD) framework, and with the goal of maintaining grassland plant communities in their current state, our findings suggest that temperate grassland plant communities are resilient to changing conditions, supporting an “accept” approach for now, in which managers allow ecological change to proceed without attempting to prevent it. Managed disturbance regimes promote grassland plant diversity and function, regardless of timing, and outcomes are not significantly affected by winter climate change, implying that immediate, drastic management adaptations may not be necessary to prevent degradation.

However, while many grassland species demonstrate resilience to stress, they are likely most vulnerable during the germination and establishment phases (Walck et al., 2010), particularly in newly restored communities. In contrast, mature, well‐established communities (i.e., old‐growth grasslands; sensu Veldman et al., 2015) may be better equipped to withstand future climate stressors. Therefore, prioritizing the successful establishment of desired communities in the short term could enhance long‐term resilience. If winter insulation from snow continues to diminish, it may be necessary to shift toward a “resist” strategy, adapting management practices to maintain disturbance regimes that promote colonization while preserving insulating cover in the winter, such as by burning only in spring, to prevent potential future degradation.

## AUTHOR CONTRIBUTIONS

J.J.H. and E.I.D. led project conceptualization, and all authors contributed to ongoing investigation. K.T.C., J.J.H., and M.A.H. curated the community data, and C.R.W. primarily curated the functional trait data. K.T.C. led formal analysis, visualization, and writing the initial manuscript draft, and all authors contributed to editing and review. E.I.D. provided lab infrastructure and supervision. Research questions, methods, and interpretation were developed in collaboration with local land managers, including representatives from Adaptive Restoration, the Nature Conservancy in Wisconsin, the Prairie Enthusiasts Empire‐Sauk Chapter, and the Wisconsin Department of Natural Resources.

## Supporting information


Appendix 1.
**Table S1.** Timing of disturbance treatments, number of winter snow manipulations, and total December, January, and February (DJF) snowfall each year of the study.
**Table S2.** Average approximate overwinter snow, litter, and total winter insulation depth after manipulations in each disturbance type and timing and winter snow manipulations combination.
**Table S3.** Coverage of functional trait data across all taxa in the herbaceous community data and hypothesized tradeoffs with stress tolerance and resource acquisition.
**Table S4.** Estimated coefficient, standard error, *P*‐value, and fixed‐effects partial *r*‐squared for the effects of disturbance type and timing, winter snow manipulations, and their interaction on various measures of community change.
**Table S5.** Estimated coefficient, standard error, *P*‐value, and fixed‐effects partial *r*‐squared for the effects of winter soil temperature on various measures of community change.
**Table S6.** Test statistics, sum of squares, *P*‐values, and fixed‐effects partial *r*‐squared for the effects of disturbance type and timing, winter snow manipulations, and their interaction on various measures of community turnover.
**Table S7.** Estimated coefficient, standard error, *P*‐value, and fixed‐effects partial *r*‐squared for the effects of the stress tolerance–resource acquisition functional trait trade‐off (PC2) and its interaction with disturbance type and timing and winter snow manipulations on change in abundance, probability of colonization, and probability of extinction.
**Table S8.** Estimated coefficient, standard error, *P*‐value, and fixed‐effects partial *r*‐squared for the effects of the stress tolerance–resource acquisition functional trait trade‐off (PC2) and its interaction with winter soil temperature on change in abundance, probability of colonization, and probability of extinction.
**Figure S1.** Rank abundance curve of the plant community across all 7 years of the study. The species codes in the legend are ordered by relative abundance.
**Figure S2.** Nonmetric multidimensional scaling (NMDS) scores along the first and second axes for increases (left) and decreases (right) in abundance over the study period.
**Figure S3.** Effects of average minimum and maximum December, January, and February (DJF) temperature on changes in the plant community, including estimated biomass (top), species richness (middle), and Shannon's diversity (bottom).
**Figure S4.** Nonmetric multidimensional scaling (NMDS) scores along the second and third axes for increases (left) and decreases (right) in abundance.
**Figure S5.** Effects of minimum and maximum December, January, and February (DJF) temperature, PC2, and their interaction on community turnover, including change in abundance (left), probability of colonization (middle), and probability of extinction (right).

## Data Availability

Data are publicly available through ScienceBase at https://doi.org/10.5066/P1ZY9K23 (Charton, 2025).
