# Supplementary material for: A functional trait perspective on restored temperate grassland responses to changing winter insulation and managed disturbance by fire
Source: Am J Bot. 2025 Oct 9;112(10):e70109. doi: 10.1002/ajb2.70109 (PMC12572695; doi:10.1002/ajb2.70109)
Supplement: Supplementary file 1 — Appendix 1. Table S1. Timing of disturbance treatments, number of winter snow manipulations, and total December, January, and February (DJF) snowfall each year of the study. Table S2. Average approximate overwinter snow, litter, and total winter insulation depth after manipulations in each disturbance type and timing and winter snow manipulations combination. Table S3. Coverage of functional trait data across all taxa in the herbaceous community data and hypothesized tradeoffs with stress tolerance and resource acquisition. Table S4. Estimated coefficient, standard error, P‐value, and fixed‐effects partial r‐squared for the effects of disturbance type and timing, winter snow manipulations, and their interaction on various measures of community change. Table S5. Estimated coefficient, standard error, P‐value, and fixed‐effects partial r‐squared for the effects of winter soil temperature on various measures of community change. Table S6. Test statistics, sum of squares, P‐values, and fixed‐effects partial r‐squared for the effects of disturbance type and timing, winter snow manipulations, and their interaction on various measures of community turnover. Table S7. Estimated coefficient, standard error, P‐value, and fixed‐effects partial r‐squared for the effects of the stress tolerance–resource acquisition functional trait trade‐off (PC2) and its interaction with disturbance type and timing and winter snow manipulations on change in abundance, probability of colonization, and probability of extinction. Table S8. Estimated coefficient, standard error, P‐value, and fixed‐effects partial r‐squared for the effects of the stress tolerance–resource acquisition functional trait trade‐off (PC2) and its interaction with winter soil temperature on change in abundance, probability of colonization, and probability of extinction. Figure S1. Rank abundance curve of the plant community across all 7 years of the study. The species codes in the legend are ordered by relative abundance. [file AJB2-112-e70109-s001.pdf]

SUPPORTING INFORMATION

Table S1. Timing of disturbance treatments, number of winter snow manipulations, and total December, January, and February (DJF) snowfall each year of the study.[1] The 30-year climate normal for DJF snowfall in south-central Wisconsin is 81.79 cm.[2] Years are defined as ending with the growing season when plant community responses were measured.

Year	Mow date	Fall burn date	Snow manipulations	Total DJF snowfall (cm)	Spring burn date
2016–2017	22 Oct 2016	27 Nov 2016	3	88.39	10 Apr 2017
2017–2018	25 Oct 2017	20 Nov 2017	3	105.16	23 Apr 2018
2018–2019	29 Oct 2018	16 Dec 2018	6	127.00	15 Apr 2019
2019–2020	22 Nov 2019	18 Dec 2019	0	114.30	22 Apr 2020
2020–2021	02 Nov 2020	05 Nov 2020	4	107.70	14 Apr 2021
2021–2022	25 Oct 2021	03 Nov 2021	2	60.96	09 Apr 2022
2022–2023	17 Oct 2022	07 Nov 2022	1	145.03	18 Apr 2023

[1] Wisconsin State Climatology Office. 2024. Divisional 12-month precipitation. Nelson Institute for Environmental Studies, University of Wisconsin-Madison, Madison, WI, USA. https://climatology.nelson.wisc.edu/wisconsin-climate-divisions/divisional-12-month-precipitation/

[2] Wisconsin State Climatology Office. 2024. Divisional climate normals. Nelson Institute for Environmental Studies, University of Wisconsin-Madison, Madison, WI, USA. https://climatology.nelson.wisc.edu/wisconsin-climate-divisions/climate-normals/

Table S2. Average approximate overwinter snow, litter, and total winter insulation depth after manipulations in each disturbance type and timing and winter snow manipulations combination.

Management seasonality treatment	Snow depth treatment	Average snow depth (cm)	Average litter depth (cm)	Average total winter insulation depth (cm)
Unmanaged control	Unmanipulated control	24.66	9.03	33.69
Unmanaged control	Reduced snow	4.37	8.50	12.87
Unmanaged control	Additional snow	49.69	12.31	62.00
Spring fire	Unmanipulated control	26.41	1.21	27.62
Spring fire	Reduced snow	3.94	1.07	5.01
Spring fire	Additional snow	50.59	1.39	51.98
Fall fire	Unmanipulated control	11.87	1.56	13.43
Fall fire	Reduced snow	2.41	1.67	4.08
Fall fire	Additional snow	36.82	3.88	40.70
Fall mow	Unmanipulated control	13.21	7.72	20.93
Fall mow	Reduced snow	3.39	9.31	12.70
Fall mow	Additional snow	37.42	7.50	44.92

Table S3. Coverage of functional trait data across all taxa in the herbaceous community data and hypothesized trade-offs with stress tolerance and resource acquisition.

Functional trait	Unit	Range	Coverage across all taxa	Coverage across total abundance	Hypothesized trade-offs
Plant height	cm	12.26–156.85	90.63%	98.94%	↓ stress tolerance<br>↑ resource acquisition[1–3]
Specific leaf area (SLA)	m^2/kg	6.61–31.77	89.06%	99.92%	↓ stress tolerance<br>↑ resource acquisition[1–4]
Leaf area	cm^2	0.37–427.63	89.06%	99.92%	↓ stress tolerance<br>↑ resource acquisition[3]
Leaf dry matter content (LDMC)	mg/g	131.49–1289.62	98.44%	98.74%	↑ stress tolerance<br>↓ resource acquisition[3]
Foliar nitrogen content (N)	%	0.73–4.12	95.31%	99.07%	↓ stress tolerance<br>↑ resource acquisition[3,4]
Leaf tissue cold tolerance (LT_{50})	°C	-9.98–-6.49	50.00%	83.33%	↑ stress tolerance<br>↓ resource acquisition[5]
Dispersule mass	mg	0.03–838.44	92.19%	99.18%	↓ stress tolerance<br>↑ resource acquisition[1–3]

[1] Westoby, M. 1998. A leaf-height-seed (LHS) plant ecology strategy scheme. *Plant and Soil* 199: 213–227. https://doi.org/10.1023/A:1004327224729

[2] Adler, P. B., R. Salguero-Gómez, A. Compagnoni, J. S. Hsu, J. Ray-Mukherjee, C. Mbeau-Ache, and M. Franco. 2013. Functional traits explain variation in plant life history strategies. *Proceedings of the National Academy of Sciences, USA* 111: 740–745. https://doi.org/10.1073/pnas.1315179111

[3] Díaz, S., J. Kattge, J. H. C. Cornelissen, I. J. Wright, S. Lavorel, S. Dray, B. Reu, et al. 2016. The global spectrum of plant form and function. *Nature* 529: 167–171. https://doi.org/10.1038/nature16489

[4] Wright, I. J., P. B. Reich, M. Westoby, D. D. Ackerly, Z. Baruch, F. Bongers, J. Cavender-Bares, et al. 2004. The worldwide leaf economics spectrum. *Nature* 428: 821–827. https://doi.org/10.1038/nature02403

[5] Raunkiær, C. 1934. The life forms of plants and statistical plant geography. Clarendon Press, Oxford, UK.

Table S4. Estimated coefficient, standard error, *P*-value, and fixed-effects partial *r*-squared for the effects of disturbance type and timing, winter snow manipulations, and their interaction on various measures of community change. Significant predictors are indicated by asterisks: *P < 0.05 and ***P < 0.001.

Response	Predictor	β	SE	P	r^2
Change in estimated biomass (cm^3)	Intercept	0.664	0.151	<0.001***	0.054
	Spring burn	-0.097	0.120	0.423	
	Fall burn	-0.102	0.120	0.400	
	Fall mow	-0.088	0.120	0.463	
	Reduced snow	0.222	0.120	0.067	
	Additional snow	-0.235	0.120	0.053	
	Spring fire × reduced snow	-0.319	0.170	0.062	
	Fall fire × reduced snow	-0.116	0.170	0.495	
	Fall mow × reduced snow	-0.145	0.170	0.396	
	Spring fire × additional snow	0.294	0.170	0.085	
	Fall fire × additional snow	0.231	0.170	0.177	
	Fall mow × additional snow	0.339	0.170	0.048*	
Change in species richness (*S*)	Intercept	0.224	0.055	<0.001***	0.095
	Spring fire	0.159	0.068	0.021*	
	Fall fire	0.113	0.068	0.100	
	Fall mow	0.148	0.068	0.031*	
	Reduced snow	0.118	0.068	0.086	
	Additional snow	0.056	0.068	0.413	
	Spring fire × reduced snow	-0.129	0.097	0.183	
	Fall fire × reduced snow	0.018	0.097	0.852	

Response	Predictor	*β*	SE	*P*	r^2
	Fall mow × reduced snow	-0.052	0.097	0.591	
	Spring fire × additional snow	-0.009	0.097	0.924	
	Fall fire × additional snow	-0.010	0.097	0.922	
	Fall mow × additional snow	-0.070	0.097	0.471	
Change in Shannon's diversity (*H′*)	Intercept	0.163	0.079	0.045*	0.055
	Spring fire	-0.103	0.095	0.285	
	Fall fire	-0.135	0.095	0.163	
	Fall mow	-0.096	0.095	0.317	
	Reduced snow	-0.052	0.083	0.53	
	Additional snow	-0.007	0.083	0.93	
	Spring fire × reduced snow	0.09	0.117	0.443	
	Fall fire × reduced snow	0.149	0.117	0.205	
	Fall mow × reduced snow	0.007	0.117	0.949	
	Spring fire × additional snow	0.202	0.117	0.086	
	Fall fire × additional snow	0.048	0.117	0.683	
	Fall mow × additional snow	-0.015	0.117	0.898	

Table S5. Estimated coefficient, standard error, *P*-value, and fixed-effects partial *r*-squared for the effects of winter soil temperature on various measures of community change. Significant predictors are indicated by asterisks: *$P < 0.05$, **$P < 0.01$, and ***$P < 0.001$.

Response	Predictor	β	SE	P	r^2
Change in estimated biomass (cm^3)	Intercept	0.659	0.144	<0.001***	0.001
	Minimum DJF temperature (°C)	0.007	0.010	0.455	
	Intercept	0.689	0.161	<0.001***	0.017
	Maximum DJF temperature (°C)	-0.009	0.012	0.436	
Change in species richness (*S*)	Intercept	0.310	0.049	<0.001***	0.010
	Minimum DJF temperature (°C)	-0.009	0.006	0.122	
	Intercept	0.401	0.066	<0.001***	0.001
	Maximum DJF temperature (°C)	-0.004	0.007	0.582	
Change in Shannon's diversity (*H′*)	Intercept	0.105	0.074	0.172	0.001
	Minimum DJF temperature (°C)	0.001	0.008	0.953	
	Intercept	0.265	0.093	0.006**	0.029
	Maximum DJF temperature (°C)	-0.019	0.009	0.033*	

Table S6. Test statistics, sum of squares, *P*-values, and fixed-effects partial *r*-squared for the effects of disturbance type and timing, winter snow manipulations, and their interaction on various measures of community turnover. Significant predictors are indicated by asterisks: *$P < 0.05$ and ***$P < 0.001$.

Response	Predictor	F	SS	P	r^2
Increases in abundance	Spring fire	3.410	0.814	<0.001***	0.016
	Fall fire	1.619	0.386	0.085	0.008
	Fall mow	1.840	0.439	0.049*	0.008
	Reduced snow	0.491	0.117	0.928	0.002
	Additional snow	1.029	0.246	0.384	0.005
	Spring fire × reduced snow	1.246	0.298	0.239	0.006
	Fall fire × reduced snow	1.117	0.267	0.324	0.005
	Fall mow × reduced snow	0.917	0.219	0.525	0.004
	Spring fire × additional snow	0.967	0.231	0.451	0.005
	Fall fire × additional snow	1.093	0.261	0.368	0.005
	Fall mow × additional snow	1.009	0.241	0.396	0.005
Decreases in abundance	Spring fire	1.737	0.442	0.079	0.007
	Fall fire	2.042	0.520	0.035*	0.008
	Fall mow	1.379	0.351	0.177	0.005
	Reduced snow	1.178	0.300	0.278	0.005
	Additional snow	0.455	0.116	0.932	0.002
	Spring fire × reduced snow	0.703	0.179	0.714	0.003
	Fall fire × reduced snow	1.238	0.315	0.251	0.005
	Fall mow × reduced snow	1.136	0.289	0.347	0.004

Response	Predictor	*F*	SS	*P*	r^2
	Spring fire × additional snow	2.012	0.512	0.047*	0.008
	Fall fire × additional snow	1.330	0.339	0.201	0.005
	Fall mow × additional snow	1.049	0.267	0.382	0.004

Table S7. Estimated coefficient, standard error, *P*-value, and fixed-effects partial *r*-squared for the effects of the stress tolerance-resource acquisition functional trait tradeoff (PC2) and its interaction with disturbance type and timing and winter snow manipulations on change in abundance, probability of colonization, and probability of extinction. Significant predictors are indicated by asterisks: **P* < 0.05 and ****P* < 0.001.

Response	Predictor	β	SE	P	r^2
Change in abundance (%)	PC2	-0.027	0.108	0.801	0.023
	Spring burn × PC2	-0.287	0.146	0.049*	
	Fall burn × PC2	0.064	0.147	0.661	
	Fall mow × PC2	-0.166	0.145	0.251	
	Snow reduction × PC2	-0.111	0.147	0.451	
	Snow addition × PC2	-0.099	0.16	0.538	
	Spring burn × Snow reduction × PC2	0.263	0.206	0.202	
	Fall burn × Snow reduction × PC2	-0.168	0.204	0.41	
	Fall mow × Snow reduction × PC2	0.248	0.206	0.228	
	Spring burn × Snow addition × PC2	0.414	0.211	0.05	
	Fall burn × Snow addition × PC2	-0.152	0.211	0.47	
	Fall mow × Snow addition × PC2	0.104	0.211	0.623	
Probability of colonization	PC2	0.412	0.109	<0.001***	0.138
	Spring burn × PC2	0.204	0.158	0.195	
	Fall burn × PC2	0.209	0.153	0.17	
	Fall mow × PC2	0.098	0.154	0.524	
	Snow reduction × PC2	0.181	0.157	0.25	

Response	Predictor	*β*	SE	*P*	r^2
	Snow addition × PC2	-0.055	0.152	0.719	
	Spring burn × Snow reduction × PC2	-0.337	0.219	0.125	
	Fall burn × Snow reduction × PC2	-0.486	0.211	0.022*	
	Fall mow × Snow reduction × PC2	-0.268	0.214	0.209	
	Spring burn × Snow addition × PC2	-0.243	0.21	0.248	
	Fall burn × Snow addition × PC2	-0.121	0.21	0.565	
	Fall mow × Snow addition × PC2	0.082	0.218	0.705	
Probability of extinction	PC2	-0.17	0.199	0.394	0.066
	Spring burn × PC2	-0.069	0.172	0.687	
	Fall burn × PC2	0.03	0.185	0.873	
	Fall mow × PC2	-0.081	0.16	0.614	
	Snow reduction × PC2	0.01	0.159	0.951	
	Snow addition × PC2	0.248	0.281	0.377	
	Spring burn × Snow reduction × PC2	0.102	0.28	0.717	
	Fall burn × Snow reduction × PC2	-0.115	0.258	0.656	
	Fall mow × Snow reduction × PC2	0.207	0.275	0.451	
	Spring burn × Snow addition × PC2	-0.207	0.249	0.404	

Response	Predictor	β	SE	P	r^2
	Fall burn × Snow addition × PC2	-0.329	0.27	0.223	
	Fall mow × Snow addition × PC2	-0.17	0.199	0.394	

Table S8. Estimated coefficient, standard error, *P*-value, and fixed-effects partial *r*-squared for the effects of the stress tolerance-resource acquisition functional trait tradeoff (PC2) and its interaction with winter soil temperature on change in abundance, probability of colonization, and probability of extinction.

Response	Predictor	β	SE	p	r^2
Change in abundance (%)	PC2	-0.136	0.297	0.647	0.019
	Minimum DJF temperature × PC2	-0.020	0.040	0.612	
	Maximum DJF temperature × PC2	-0.028	0.035	0.424	
Probability of colonization	PC2	0.188	0.237	0.426	0.127
	Minimum DJF temperature × PC2	-0.037	0.033	0.260	
	Maximum DJF temperature × PC2	0.034	0.030	0.254	
Probability of extinction	PC2	-0.255	0.356	0.475	0.010
	Minimum DJF temperature × PC2	-0.056	0.050	0.264	
	Maximum DJF temperature × PC2	0.017	0.044	0.698	

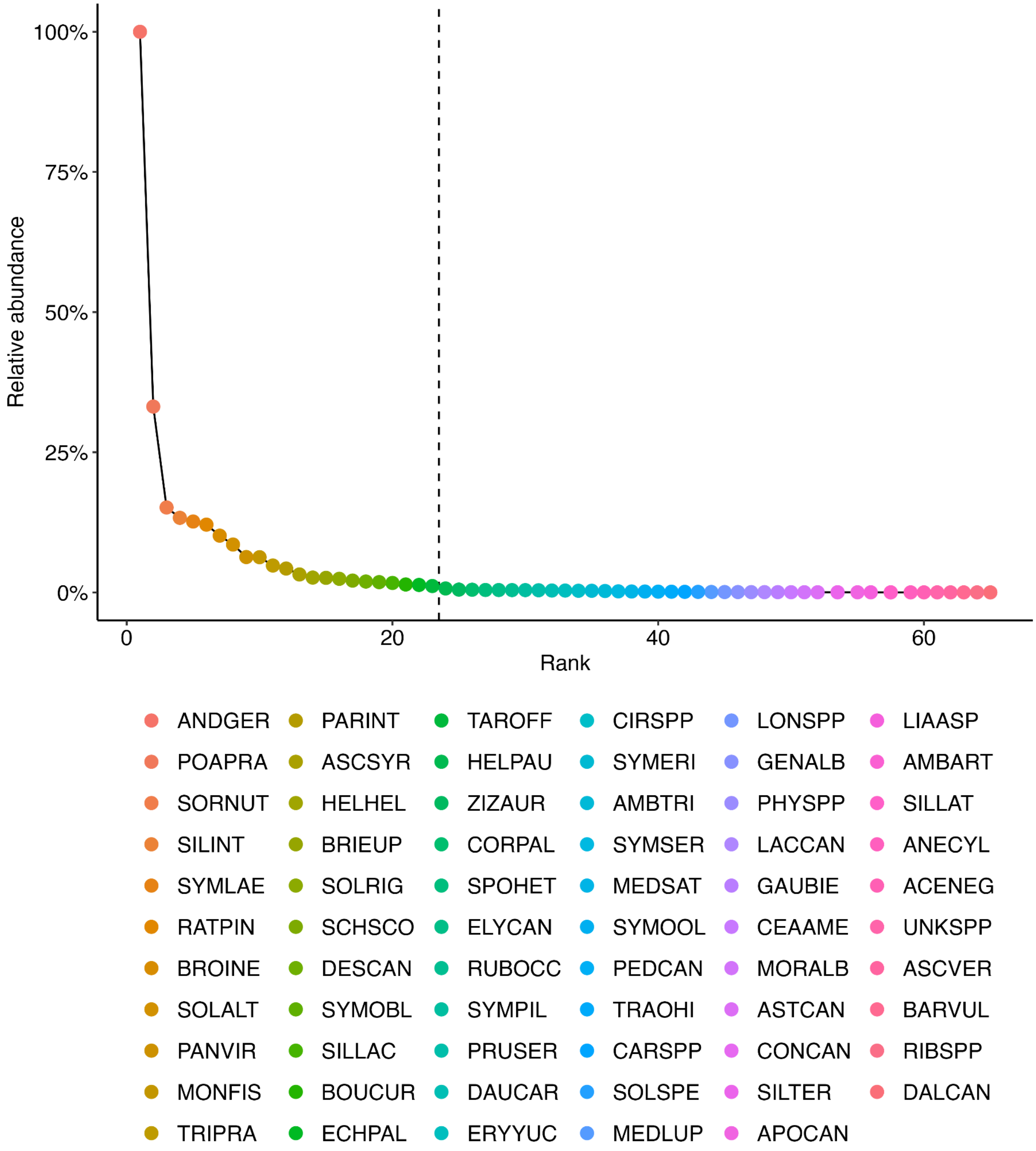

Figures S1. Rank abundance curve of the plant community across all 7 years of the study. The species codes in the legend are ordered by relative abundance. The vertical dashed line represents the cut-off between species with >1% relative abundance (left) and <1% relative abundance (right). ANDGER = *Andropogon gerardii* Vitman, POAPRA = *Poa pratensis* L., SORNUT = *Sorghastrum nutans* (L.) Nash, SILINT = *Silphium integrifolium* Michx., SYMLAE = *Symphyotrichum laeve* (L.) Á. Löve & D. Löve, RATPIN = *Ratibida pinnata* (Vent.) Barnhart, BROINE = *Bromus inermis* Leyss., SOLALT = *Solidago altissima* L., PANVIR = *Panicum*

virgatum L., MONFIS = *Monarda fistulosa* Beck, TRIPRA = *Trifolium pratense* L., PARINT = *Parthenium integrifolium* Britton, ASCSYR = *Asclepias syriaca* L., HELHEL = *Heliopsis helianthoides* (L.) Sweet, BRIEUP = *Brickellia eupatorioides* (L.) Shinners, SOLRIG = *Solidago rigida* L., SCHSCO = *Schizachyrium scoparium* (Nash) E.P. Bicknell, DESCAN = *Desmodium canadense* (L.) DC., SYMOBL = *Symphyotrichum oblongifolium* (Nutt.) G.L. Nesom, SILLAC = *Silphium laciniatum* L., BOUCUR = *Bouteloua curtipendula* (Michx.) Torr., ECHPAL = *Echinacea pallida* (Nutt.) Nutt., TAROFF = *Taraxacum officinale* F.H. Wigg., HELPAU = *Helianthus pauciflorus* Nutt., ZIZAUR = *Zizia aurea* (L.) W.D.J. Koch, CORPAL = *Coreopsis palmata* Nutt., SPOHET = *Sporobolus heterolepis* (A. Gray) A. Gray, ELYCAN = *Elymus canadensis* L., RUBOCC = *Rubus occidentalis* L., SYMPIL = *Symphyotrichum pilosum* (Willd.) G.L. Nesom, PRUSER = *Prunus serotina* C. Mohr, DAUCAR = *Daucus carota* L., ERYYUC = *Eryngium yuccifolium* Michx., CIRSPP = *Cirsium* Mill. or *Carduus* L. spp., SYMERI = *Symphyotrichum ericoides* (L.) G.L. Nesom, AMBTRI = *Ambrosia trifida* L., SYMSER = *Symphyotrichum sericeum* (Vent.) G.L. Nesom, MEDSAT = *Medicago sativa* L., SYMOOL = *Symphyotrichum oolentangiense* (Riddell) G.L. Nesom, PEDCAN = *Pedicularis canadensis* L., TRAOHI = *Tradescantia ohiensis* Raf., CARSPP = *Carex* L. spp., SOLSPE = *Solidago speciosa* Nutt., MEDLUP = *Medicago lupulina* L., LONSPP = *Lonicera* L. spp., GENALB = *Gentiana alba* Muhl. ex Nutt., PHYSPP = *Physalis* L. spp., LACCAN = *Lactuca canadensis* L., GAUBIE = *Gaura biennis* (Small) W.L. Wagner & Hoch, CEAAME = *Ceanothus americanus* L., MORALB = *Morus alba* L., ASTCAN = *Astragalus canadensis* L., CONCAN = *Conyza canadensis* (L.) Cronquist, SILTER = *Silphium terebinthinaceum* Jacq., APOCAN = *Apocynum cannabinum* L., LIAASP = *Liatris aspera* Michx., AMBART = *Ambrosia artemisiifolia* L., SILLAT = *Silene latifolia* Poir., ANECYL = *Anemone cylindrica* A. Gray, ACENEG = *Acer negundo* L., UNKSPP = Unknown taxon that is different from all others listed here, ASCVER = *Asclepias verticillata* L., BARVUL = *Barbarea vulgaris* W.T. Aiton, RIBSPP = *Ribes* L. spp., DALCAN = *Dalea candida* Michx. ex Willd.

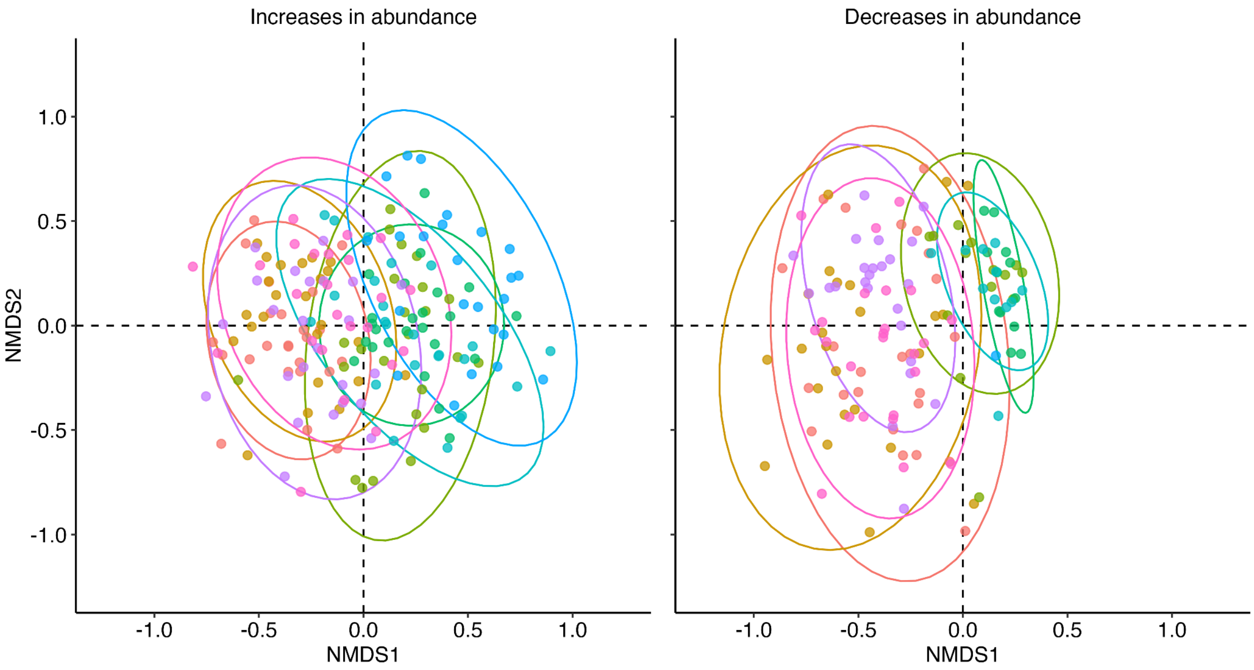

Figure S2. Nonmetric multidimensional scaling (NMDS) scores along the first and second axes for increases (left) and decreases (right) in abundance over the study period. Points represent individual subplots and are colored by experimental treatment block. Ellipses represent the 95% confidence interval for each block.

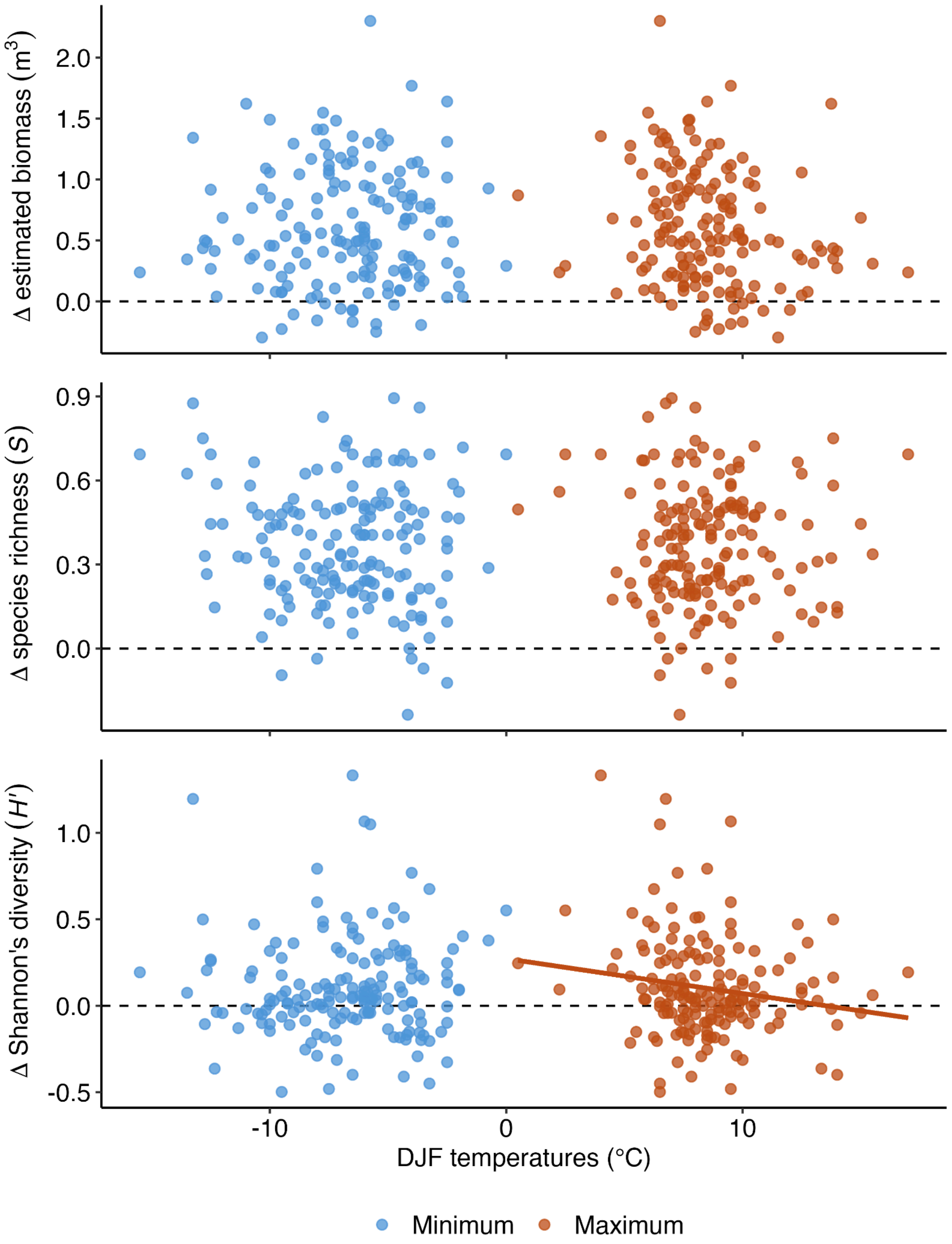

Figure S3. Effects of average minimum and maximum December, January, and February (DJF) temperature on changes in the plant community, including estimated biomass (top), species richness (middle), and Shannon's diversity (bottom). Points represent individual subplots, and trendlines indicate significant relationships between temperature and community changes.

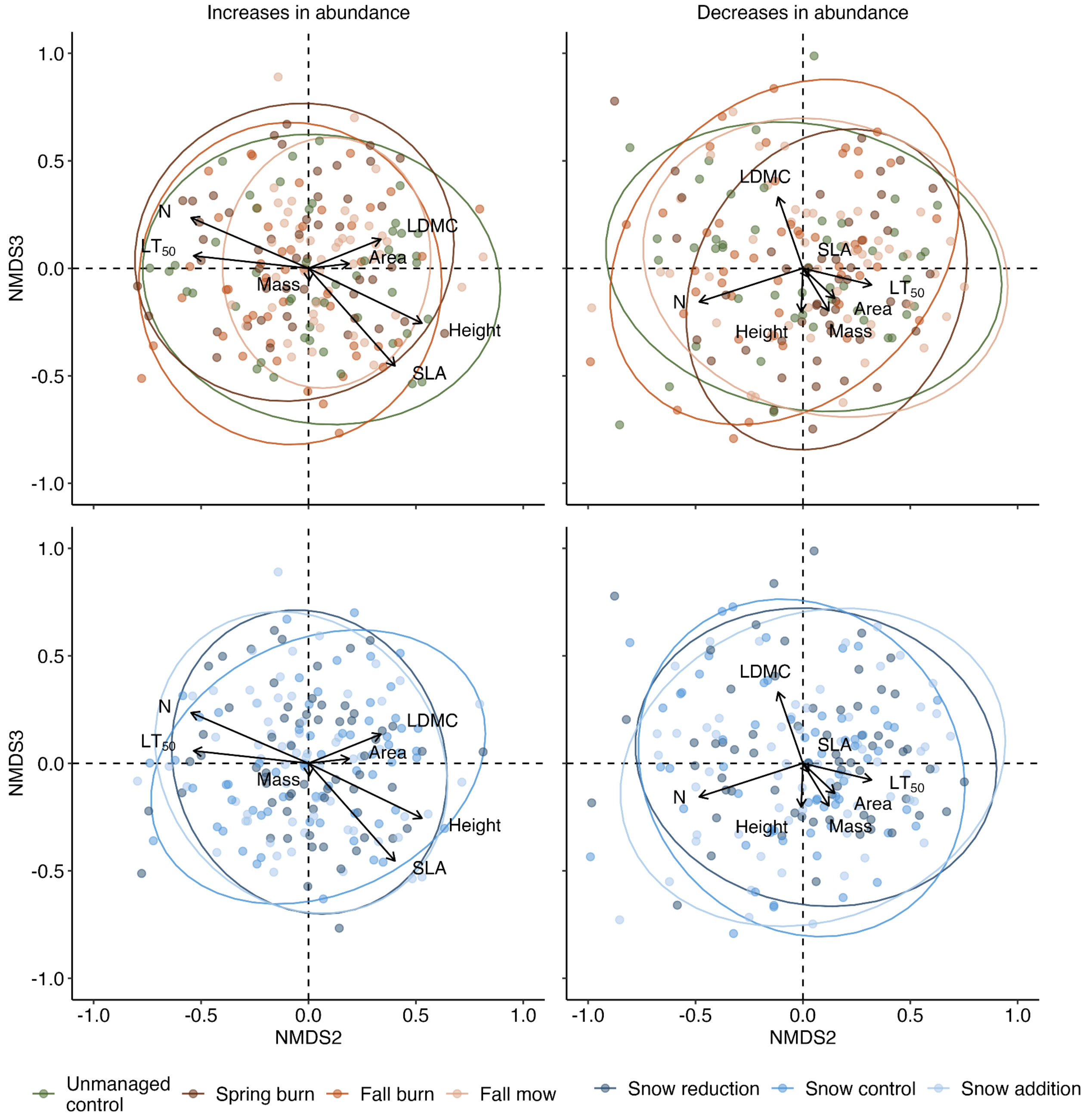

Figure S4. Nonmetric multidimensional scaling (NMDS) scores along the second and third axes for increases (left) and decreases (right) in abundance. Points represent individual subplots and are colored by disturbance type and timing (top) or winter snow manipulations (bottom). Ellipses represent the 95% confidence interval for each treatment. Vectors represent the loading of each trait based on community-weighted means. Height = plant height, SLA = specific leaf area, Area = leaf area, LDMC = leaf dry matter content, N = foliar nitrogen content, LT_{50} = leaf tissue cold tolerance, Mass = dispersule mass.

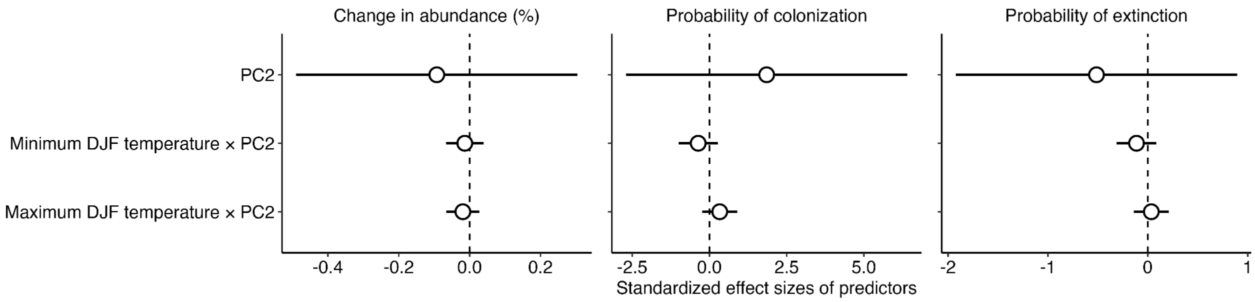

Figure S5. Effects of minimum and maximum December, January, and February (DJF) temperature, PC2, and their interaction on community turnover, including change in abundance (left), probability of colonization (middle), and probability of extinction (right). Points represent average standardized effect sizes, and error bars represent 95% confidence intervals. Note axes are on different scales.
